# Supplementary material for: Effects of Vitamin B12 Supplementation on Cognitive Function, Depressive Symptoms, and Fatigue: A Systematic Review, Meta-Analysis, and Meta-Regression
Source: Nutrients. 2021 Mar 12;13(3):923. doi: 10.3390/nu13030923 (PMC8000524; doi:10.3390/nu13030923)
Supplement: Supplementary file 1 [file nutrients-13-00923-s001.pdf]

Supplement Table S1: Search strategies

Ovid MEDLINE®

#	Searches	Results
1	exp Vitamin B 12/ or (vitamin B12 or vitamin b-12 or hydroxocobalamin or cyanocobalamin or b12 or b 12 or Docibin or Ducobee or cobalamin or methylcobalamin or adenosylcobalamin or mecobalamin or ydroxycobalamin).ti,ab.	36,306
2	exp Cognition/ or exp Fatigue/ or exp Lethargy/ or exp sleepiness/ or exp Attention/ or exp Depression/ or (fatigue or tiredness or weariness or exhaustion or attentiveness or listlessness or lethargy or cognition or avolition or sluggishness or sleepiness or drowsiness or concentration or focus or attention or forgetfulness or mood or depressed or depressive or depression*).ti,ab. or (cognitive adj1 (impairment or function or performance or decline)).ti,ab.	2,950,374
3	(randomized controlled trial or controlled clinical trial).pt. or randomized.ti,ab. or randomised.ti,ab. or placebo.ti,ab. or drug therapy.fs. or randomly.ti,ab. or trial.ti,ab. or groups.ti,ab.	4,795,717
4	1 and 2 and 3	1,805
5	4 not (animals not humans).sh.	1,627

Embase®

#	Searches	Results
1	'cyanocobalamin'/exp OR 'hydroxocobalamin'/exp OR 'vitamin b12':ti,ab OR 'vitamin b-12':ti,ab OR hydroxocobalamin:ti,ab OR cyanocobalamin:ti,ab OR 'b12':ti,ab OR 'b 12':ti,ab OR docibin:ti,ab OR ducobee:ti,ab OR cobalamin:ti,ab OR methylcobalamin:ti,ab OR adenosylcobalamin:ti,ab OR mecobalamin:ti,ab OR hydroxycobalamin:ti,ab	57,879
2	'cognition'/exp OR 'fatigue'/exp OR 'lethargy'/exp OR 'listlessness'/exp OR 'somnolence'/exp OR 'drowsiness'/exp OR 'depression'/exp OR 'attention'/exp OR 'cognitive defect'/exp OR 'memory'/exp OR fatigue:ti,ab OR tiredness:ti,ab OR weariness:ti,ab OR exhaustion:ti,ab OR attentiveness:ti,ab OR listlessness:ti,ab OR lethargy:ti,ab OR cognition:ti,ab OR avolition:ti,ab OR sluggishness:ti,ab OR sleepiness:ti,ab OR drowsiness:ti,ab OR concentration:ti,ab OR focus:ti,ab OR attention:ti,ab OR forgetfulness:ti,ab OR mood:ti,ab OR depressed:ti,ab OR depressive:ti,ab OR depression*:ti,ab OR memory:ti,ab OR ((cognitive NEAR/1 (impairment OR function OR performance OR decline)):ti,ab)	5,695,399
3	'crossover procedure':de OR 'double-blind procedure':de OR 'randomized controlled trial':de OR 'single-blind procedure':de OR random*:de,ab,ti OR factorial*:de,ab,ti OR crossover*:de,ab,ti OR ((cross NEXT/1 over*):de,ab,ti) OR placebo*:de,ab,ti OR ((doubl* NEAR/1 blind*):de,ab,ti) OR ((singl* NEAR/1 blind*):de,ab,ti) OR assign*:de,ab,ti OR allocat*:de,ab,ti OR volunteer*:de,ab,ti	2,567,179
4	#1 AND #2 AND #3	2,070
5	#1 AND #2 AND #3 NOT [conference abstract]/lim NOT ([animals]/lim NOT [humans]/lim)	1,647

PsycINFO

#	Searches	Results
1	TI ((vitamin B12 OR vitamin b-12 OR hydroxocobalamin OR cyanocobalamin OR b12 OR b 12 OR Docibin OR Ducobee OR cobalamin OR methylcobalamin OR adenosylcobalamin OR mecobalamin OR hydroxycobalamin)) OR AB ((vitamin B12 OR vitamin b-12 OR hydroxocobalamin OR cyanocobalamin OR b12 OR b 12 OR Docibin OR Ducobee OR cobalamin OR methylcobalamin OR adenosylcobalamin OR mecobalamin OR hydroxycobalamin))	8,842
2	TI ((fatigue OR tiredness OR weariness OR exhaustion OR attentiveness OR listlessness OR lethargy OR cognition OR avolition OR sluggishness OR sleepiness OR drowsiness OR concentration OR focus OR attention OR forgetfulness OR mood OR depressed OR depressive OR depression* OR memory) OR (cognitive N1 (impairment OR function OR performance OR decline))) OR AB ((fatigue OR tiredness OR weariness OR exhaustion OR attentiveness OR listlessness OR lethargy OR cognition OR avolition OR sluggishness OR sleepiness OR drowsiness OR concentration OR focus OR attention OR forgetfulness OR mood OR depressed OR depressive OR depression* OR memory) OR (cognitive N1 (impairment OR function OR performance OR decline))) OR (DE "Cognition" OR DE "Fatigue" OR DE "Sleepiness" OR DE "Attention" OR DE "Major Depression" OR DE "Memory" OR DE "Forgetting")	1,228,483
3	TI (placebo* OR random* OR "comparative stud*" OR clinical N3 trial* OR research N3 design OR evaluat* N3 stud* OR prospectiv* N3 stud* OR (singl* OR doubl* OR trebl* OR tripl*) N3 (blind* OR mask*)) OR AB (placebo* OR random* OR "comparative stud*" OR clinical N3 trial* OR research N3 design OR evaluat* N3 stud* OR prospectiv* N3 stud* OR (singl* OR doubl* OR trebl* OR tripl*) N3 (blind* OR mask*)) OR (DE "Treatment Outcomes" OR DE "Treatment Effectiveness Evaluation" OR DE "Placebo" OR DE "Followup Studies")	422,395
4	S1 AND S2 AND S3	717

Cochrane Library

#	Searches	Results
1	("vitamin B12" OR "vitamin b-12" OR hydroxocobalamin OR cyanocobalamin OR "b12" OR "b 12" OR Docibin OR Ducobee OR cobalamin OR methylcobalamin OR adenosylcobalamin OR mecobalamin OR hydroxycobalamin):ti,ab,kw	3,601
2	(fatigue OR tiredness OR weariness OR exhaustion OR attentiveness OR listlessness OR lethargy OR cognition OR avolition OR sluggishness OR sleepiness OR drowsiness OR concentration OR focus OR attention OR forgetfulness OR mood OR depressed OR depressive OR depression* OR memory OR (cognitive NEAR/1 (impairment OR f ti OR f OR d li))) ti b k	280,546
3	#1 AND #2	1,043

Scopus

#	Searches	Results
5	(TITLE-ABS-KEY ("vitamin B12" OR "vitamin b-12" OR hydroxocobalamin OR cyanocobalamin OR b12 OR "b 12" OR Docibin OR Ducobee OR cobalamin OR methylcobalamin OR adenosylcobalamin OR mecobalamin OR hydroxycobalamin)) AND (TITLE-ABS-KEY (fatigue OR tiredness OR weariness OR exhaustion OR attentiveness OR listlessness OR lethargy OR cognition OR avolition OR sluggishness OR sleepiness OR drowsiness OR {concentration} OR {focus} OR attention OR forgetfulness OR mood OR depressed OR depressive OR depression OR memory OR (cognitive W/1 (impairment OR function OR performance OR decline)))) AND (TITLE-ABS-KEY (random* OR crossover* OR crossingover* OR factorial* OR placebo* OR volunteer* OR control* OR prospectiv* OR volunteer* OR ((singl* OR doubl* OR trebl* OR tripl*) AND (blind* OR mask)) OR (clin* AND trial*)) OR TITLE-ABS-KEY ("randomized controlled trial" OR randomization OR "controlled study" OR "multicenter study" OR "phase 3 clinical trial" OR "phase 4 clinical trial" OR "double blind procedure" OR "single blind procedure" OR "randomized controlled trial" OR "controlled clinical trial" OR "random allocation" OR "double blind method" OR "single blind method" OR "clinical trial" OR placebo OR "research design" OR "comparative study" OR "evaluation studies" OR "follow up studies" OR "prospective studies")) AND NOT (TITLE-ABS-KEY ((animal OR ewe OR lamb OR rat OR mouse OR mice OR rodent OR horse OR equine OR cow OR bacteria* OR "E.coli" OR "E. coli" OR column OR extraction OR dog OR canine OR pig) AND NOT (human OR patient OR children OR older* OR elder* OR people OR women OR men OR girl OR boy OR addict OR infant OR toddler OR adult)))	5,216

Supplement Table S2: Clinical tests used in the included studies

Test	Description	Author, year	References
Cognitive Executive			
Block design	Rearrange different colored blocks (white, red, or both white and red) to match a pattern (7 templates).	Lewerin, 2005 [79]	Wechsler D. The measurement and appraisal of adult intelligence. Baltimore, MD: Williams & Wilkins; 1958.
Category Fluency Test (CFT)	To name animals, vegetables and fruits in 1 min each	Kwok, 2017 [74]<br>Kwok, 2020 [86]	Acevedo A, Loewenstein DA, Barker WW, Harwood DG, Luis C, Bravo M, Hurwitz DA, Aguero H, Greenfield L, Duara R. Category Fluency Test: Normative data for English- and Spanishspeaking elderly. J Int Neuropsychol Soc 2000; 6: 760-9.
Category Word Fluency Test	Name as many words as possible of a specific category (during a 1 minute period).	McMahon, 2006 [80]	Lezak MD. Neuropsychological assessment. 3rd ed. New York: Oxford University Press, 1995.
Clock Drawing Test (CDT)	Draw the face of a clock, mark in the hours and then draw the hands to indicate a specified time (for example, 10 past 10).	Ford, 2010 [77]	Brodaty H, Moore CM. The Clock Drawing Test for dementia of the Alzheimer's type: a comparison of three scoring methods in a memory disorders clinic. Int J Geriatr Psychiatry 1997;12:619–627.
Complex Figure of Rey, Copy	Copy the complex figure of Rey (a standard design of shapes and lines) from an example.	Eussen, 2006 [87]	Visser RSH. Manual of the Complex Figure Test. Lisse, Netherlands: Swets & Zeitlinger, 1985.
Controlled Oral Word Association Test (COWAT)	Name as many words as possible starting with a particular letter (during a 1 minute period, 3 letters were given).	Kwok, 2017 [74]<br>Kwok, 2020 [86]<br>McMahon, 2006 [80]	Patterson J. Controlled Oral Word Association Test. In: Kreutzer J, DeLuca J, Caplan B, eds. Encyclopedia of Clinical Neuropsychology. New York: Springer; 2011. pp. 703-6.<br>Benton A, Hamsher K. Multilingual Aphasia Examination. Iowa City, Iowa: AJA, 1989.
Figure Classification	Mark the figure that is different in a set of 5 figures.	Lewerin, 2005 [79]	Thurstone LL. Primary mental abilities. Psychometric monographs. Chicago, IL: University of Chicago Press,1938:5.

Test	Description	Author, year	References
Motor Planning 3	Press 1 of 3 buttons adjacent to a lit button as quickly as possible.	Eussen, 2006 [87]	Houx P. Cognitive aging and health-related factors. Maastricht, Netherlands: Maastricht University, 1991:113–21.
Raven's Progressive Matrices	Identify the missing element that completes a pattern.	Eussen, 2006 [87]<br>McMahon, 2006 [80]	Raven J. Guide to using the Coloured Progressive Matrices. London, United Kingdom: HK Lewis, 1965.
Similarities Wechsler Adult Intelligence Scale (WAIS)	Describe the similarities between 5 pairs of nouns.	Eussen, 2006 [87]	Wechsler D. Manual for the Wechsler Adult Intelligence Scale—revised. New York, NY: Psychological Corporation, 1981.
Stroop Color-Word Test<br>Stroop Color-Word Test Abridged	Task 1: Read the color name given in black ink.<br>Task 2: Name the colored rectangles.<br>Task 3: Name the ink color of the printed words rather than the word	Eussen, 2006 [87]<br>van Uffelen, 2008 [83]	Stroop J. Studies of interference in serial verbal reactions. J Exp Psychol 1935;18:643–662.<br>Klein M, Ponds RW, Houx PJ, et al. Effect of test duration on age-related differences in Stroop interference. J Clin Exp Neuropsychol 1997;19:77–82.
Synonyms	Select from among 5 words a synonym for a given word.	Lewerin, 2005 [79]	Dureman I, Salde H. Psykometriska och experimentalpsykologiska metoder för klinisk tillampning. (Psychometric and experimental psychological methods for clinical application.) Stockholm, Sweden: Almqvist & Wiksell, 1959 (in Swedish).
Trail Making Test	Part A: Connect randomly placed numbers (1-25) with a line as fast as possible in numeric order.<br>Part B: Connect randomly placed numbers (1-13) and letters (A-L) alternated with a line as fast as possible in numeric and alphabetical order.	Eussen, 2006 [87] (Part B)<br>van der Zwaluw, 2014 [82] (Part B/Part A)	Reitan R. Validity of the Trail Making Test as an indicator of organic brain damage. Percept Mot Skills 1958;8:271– 6.
Verbal Fluency Test (VFT)	Name as many words as possible starting with a particular letter (during a 1 minute period, 3 letters were given).	van Uffelen, 2008 [83]<br>van der Zwaluw, 2014 [82]	Lezak MD. Neuropsychological assessment. 4th ed. New York: Oxford University Press, 2004.
Verbal Fluency Test (VFT), Animals Named	Name as many animals as possible (during a 1 minute period).	Eussen, 2006 [87]<br>Dangour, 2015 [72]	Luteijn F vdPF. Handleiding Groninger Intelligentietest. (Manual Groningen Intelligence Test.) Lisse, Netherlands: Swets and Zeitlinger, 1983.<br>Goodglass H, Kaplan E. Assessment of dysphasia and

Test	Description	Author, year	References
			related disorders. Philadelphia: Lea and Febiger; 1983.
Verbal Fluency Test (VFT), Letters	List as many nouns beginning with the letter P or G as possible (during a 2 minute period).	Eussen, 2006 [87]	Luteijn F vdPF. Handleiding Groninger Intelligentietest. (Manual Groningen Intelligence Test.) Lisse, Netherlands: Swets and Zeitlinger, 1983.
Cognitive Global			
Alzheimer's Disease Assessment Scale (ADAS), Cognitive Subscale	11-item version of the ADAS-cog measuring a range of cognitive functions, including memory, language, praxis, and attention.	Ford, 2010 [77]	Rosen WG, Mohs RC, Davis KL. A new rating scale for Alzheimer's disease. Am J Psychiatry 1984;141:1356 – 1364.
Cambridge Cognitive Examination (CAMCOG)	60-item scale assessing a broad range of cognitive functions.	Hvas, 2004 [73]	Roth, M., Tym, E., Mountjoy, C.Q., Huppert, F.A., Hendrie, H., Verma, S.et al., 1986. CAMDEX. A standardised instrument for the diagnosis of mental disorder in the elderly with special reference to the early detection of dementia. Br. J. Psychiatry 149, 698– 709.
Clinical Dementia Rating (CDR), Sum of Boxes	Numeric scale used to quantify the severity of symptoms of dementia (six domains: memory, orientation, judgement and problem solving, community affairs, home and hobbies, and personal care).	Kwok, 2020 [86]	Morris JC. 1993. The Clinical Dementia Rating (CDR): current version and scoring rules. Neurology 43: 2412–2414.
Mini-Mental State Examination (MMSE)	11-item questionnaire measuring general cognitive function (5 domains: (1) orientation; (2) registration; (3) attention and calculation; (4) recall; (5) language).	Hvas, 2004 [73]<br>McMahon, 2006 [80]<br>van Uffelen, 2008 [83]<br>Ford, 2010 [77]<br>van der Zwaluw, 2014 [82]	Folstein MF, Folstein SE, McHugh PR. 1975. 'Mini-mental state'. A practical method for grading the cognitive state of patients for the clinician. J Psychiatr Res 12: 189–198.
Neuropsychological Test Battery, Total	executive (COWAT, CFT), memory (ISLT, CPAL), speed (SRT, CRT) -> average of the z-scores	Kwok, 2017 [74]	Shen JH, Shen Q, Yu H, Lai JS, Beaumont JL, Zhang Z, et al. Validation of an<br>Alzheimer's disease assessment battery in Asian participants with mild to<br>moderate Alzheimer's disease. Am J Neurodegener Dis 2014;3:158e69.

Test	Description	Author, year	References
Telephone Interview for Cognitive Status–Modified (TICS-M) Total Cognitive Functioning Score	13-item interview (modified version) assessing general cognition (4 domains: (1) orientation; (2) registration, recent memory and delayed recall (memory); (3) attention/calculation; (4) semantic memory, comprehension and repetition (language)).	Walker, 2012 [84]	de Jager CA, Budge M, Clarke R. Utility of TICS-M for the assessment of cognitive function in older adults. Int J Geriatr Psychiatry 2003;18: 318–24.
Cognitive Memory			
12 Words Learning Test, 15 min	15 minutes after completion of the fourth learning trial. Recall as many words as possible.	Hvas, 2004 [73]	Nielsen, H., Lolk, A., Krag-Sørensen, P., 1995. Normative data for eight neuropsychological tests, gathered from a random sample of Danes aged 64–83 years. Nordisk Psykologi 47, 241– 255.
12 Words Learning Test, Immediate Recall	12 words will be read aloud at a rate of one word per second. Say as many of the words as possible in any order.	Hvas, 2004 [73]	Nielsen, H., Lolk, A., Krag-Sørensen, P., 1995. Normative data for eight neuropsychological tests, gathered from a random sample of Danes aged 64–83 years. Nordisk Psykologi 47, 241– 255.
15 Word Learning Test, delayed recall<br>Rey Auditory Verbal Learning Test (RAVLT), Trial 7	Recall the words of the 15-word learning test in any order after 20 min.	Eussen, 2006 [87]<br>McMahon, 2006 [80]	Saan RJ, Deelman BG. De nieuwe 15-woordentest (A en B) een handleiding. (New 15-words test (A and B) a manual). Lisse, Netherlands: Swets & Zeitlinger, 1986.<br>Lezak MD. Neuropsychological assessment. 3rd ed. New York: Oxford University Press, 1995.
15 Word Learning Test, Immediate Recall<br>Rey Auditory Verbal Learning Test (RAVLT), Trial 1-5<br>Auditory Verbal Learning Test (AVLT), Trial 1-5<br>Rey Auditory Verbal Learning Test (RAVLT), Immediate Recall	Read 15 words 5 times and recall words in between readings in any order.	Eussen, 2006 [87]<br>McMahon, 2006 [80]<br>van Uffelen, 2008 [83]<br>van der Zwaluw, 2014 [82]	Saan RJ, Deelman BG. De nieuwe 15-woordentest (A en B) een handleiding. (New 15-words test (A and B) a manual). Lisse, Netherlands: Swets & Zeitlinger, 1986.<br>Lezak MD. Neuropsychological assessment. 3rd ed. New York: Oxford University Press, 1995.<br>Rey A. L'examen clinique en psychologie. Paris: Presses Universitaires de France, 1964.<br>Brand N, Jolles J. Learning and retrieval rate of words presented auditorily and visually. J Gen Psychol 1985; 112:201–210.

Test	Description	Author, year	References
15 Word Learning Test, recognition<br>Rey Auditory Verbal Learning Test (RAVLT), Recognition	Recognize the original 15 words of 30 words read.	Eussen, 2006 [87]<br>van der Zwaluw, 2014 [82]	Saan RJ, Deelman BG. De nieuwe 15-woordentest (A en B) een handleiding. (New 15-words test (A and B) a manual). Lisse, Netherlands: Swets & Zeitlinger, 1986.<br>Brand N, Jolles J. Learning and retrieval rate of words presented auditorily and visually. J Gen Psychol 1985; 112:201–210.
Auditory Verbal Learning Test (AVLT), Trial 6	After five repetitions of free-recall, a second “interference” list (List B) will be presented in the same manner. Recall as many words from List B as possible. After the interference trial, recall immediately the words from List A.	van Uffelen, 2008 [83]	Rey A. L’examen clinique en psychologie. Paris: Presses Universitaires de France, 1964.
California Verbal Learning Test (CVLT), Delayed Recall<br>California Verbal Learning Test (CVLT), A long-delay free recall	Recall the words of the 16-word learning test in any order after 20 min.	Dangour, 2015 [72]<br>Ford, 2010 [77]	Delis DC, Kramer JH, Kaplan E, Ober BA. California Verbal Learning Test. New York: Psychological Corporation; 1987.<br>Delis DC, Kramer JH, Kaplan E, Ober BA. California Verbal Learning Test, second edition, adult version. The Psychological Corporation; 2000.
California Verbal Learning Test (CVLT), Trial 1-3	Read 16 words 3 times and recall words in between readings in any order.	Dangour, 2015 [72]	Delis DC, Kramer JH, Kaplan E, Ober BA. California Verbal Learning Test. New York: Psychological Corporation; 1987.
California Verbal Learning Test (CVLT), Trial 1-5	Read 16 words 5 times and recall words in between readings in any order.	Ford, 2010 [77]	Delis DC, Kramer JH, Kaplan E, Ober BA. California Verbal Learning Test, second edition, adult version. The Psychological Corporation; 2000.
Complex Figure of Rey, Delayed Recall	Draw the complex figure of Rey (a standard design of shapes and lines) without the example 30 min after seeing the copy	Eussen, 2006 [87]	Visser RSH. Manual of the Complex Figure Test. Lisse, Netherlands: Swets & Zeitlinger, 1985.
Complex Figure of Rey, Immediate Recall	Draw the complex figure of Rey (a standard design of shapes and lines) without the example immediately after the copy	Eussen, 2006 [87]	Visser RSH. Manual of the Complex Figure Test. Lisse, Netherlands: Swets & Zeitlinger, 1985.
Continuous Paired Associates Learning (CPAL)	learn a series of associations between a set of simple shapes and their associated location	Kwok, 2017 [74]<br>Kwok, 2020 [86]	Bernbach, Harley A., and Gordon H. Bower. "Confidence ratings in continuous paired-associate

Test	Description	Author, year	References
			learning." Psychonomic Science 21.4 (1970): 252-253.
Digit Cancellation Test (DCT)	Detect and pencil out each target digit (1 digit for the first matrix, 2 for the second, and 3 for the third; each matrix within a 45 sec period).	Ford, 2010 [77]	Spinnler H, Tognoni G. Standardizzazione e Taratura Italiana di Test Neuropsicologici. Ital J Neurol Sci 1987; 6(suppl 8):47–50.
Digit Span Backward	Repeat a string of digits in reverse order	Lewerin, 2005 [79]<br>Eussen, 2006 [87]<br>van der Zwaluw, 2014 [82]	Wechsler D. A standardized memory scale for clinical use. J Psychol 1945;19:87–95.<br>Wechsler D. Wechsler Memory Scale—revised manual. San Antonio, TX: Psychological Corporation, 1987.<br>Wechsler D. Manual for the Adult Intelligence Scale–Revised. New York: Psychological Corporation; 1981.
Digit Span Forward	Repeat a string of digits in original order	Lewerin, 2005 [79]<br>Eussen, 2006 [87]<br>van der Zwaluw, 2014 [82]	Wechsler D. A standardized memory scale for clinical use. J Psychol 1945;19:87–95.<br>Wechsler D. Wechsler Memory Scale—revised manual. San Antonio, TX: Psychological Corporation, 1987.<br>Wechsler D. Manual for the Adult Intelligence Scale–Revised. New York: Psychological Corporation; 1981.
International Shopping List Test (ISLT)	The International Shopping List Test (ISLT) is a four trial (three learning trials and one delayed recall trial) verbal list learning test in which individuals are instructed to remember a list of 12 items that they need to obtain from their local store (common shopping list items) (3x immediate, 30min delayed)	Kwok, 2017 [74]<br>Kwok, 2020 [86]	Lim, Y. Y., Prang, K. H., Cysique, L., Pietrzak, R. H., Snyder, P. J., & Maruff, P. (2009). A method for cross-cultural adaptation of a verbal memory assessment.<br>Behavior Research Methods, 41 (4), 1190–1200.
Telephone Interview for Cognitive Status–Modified (TICS-M), Delayed Recall	Subtest of TICS-m with a 10-item non-semantically related word list. After 5 min: recall as many words as possible.	Walker, 2012 [84]	de Jager CA, Budge M, Clarke R. Utility of TICS-M for the assessment of cognitive function in older adults. Int J Geriatr Psychiatry 2003;18: 318–24.
Telephone Interview for Cognitive Status–Modified (TICS-M), Immediate Recall	Subtest of TICS-m with a 10-item non-semantically related word list. Recall as many words as possible.	Walker, 2012 [84]	de Jager CA, Budge M, Clarke R. Utility of TICS-M for the assessment of cognitive function in older adults. Int J Geriatr Psychiatry 2003;18: 318–24.

Test	Description	Author, year	References
Thurstone's Picture Memory Test	28 pictures will be shown consecutively at a rate of every 5 sec. Identify the picture among 4 similar pictures.	Lewerin, 2005 [79]	Thurstone LL. Primary mental abilities. Psychometric monographs. Chicago, IL: University of Chicago Press,1938:5.
Visual Reproduction	4 drawings will be shown to remember. Reproduce all drawings.	Lewerin, 2005 [79]	Wechsler D. A standardized memory scale for clinical use. J Psychol 1945;19:87–95.
Wechsler Paragraph Recall Test	Immediate verbal recall of a standard paragraph length passage of text.	McMahon, 2006 [80]	Wechsler D. Wechsler Memory Scale: manual. 3rd ed. San Antonio, Tex.: Psychological Corporation, 1997.
Cognitive Speed			
Digit Symbol	Replace digits with symbols according to the existing code during a 90 sec period.	Lewerin, 2005 [79]	Wechsler D. The measurement and appraisal of adult intelligence. Baltimore, MD: Williams & Wilkins; 1958.
Digit Symbol Substitution Test (DSST)	Nine symbols corresponding with nine digits. Draw symbols corresponding to the digits below numbered boxes during a 90 sec period.	van Uffelen, 2008 [83]	Uiterwijk JM. WAIS-III-NL/V. Lisse: Swets & Zeitlinger, 2001.
Finger Tapping	Press a single button as many times as possible within 30 sec.	Eussen, 2006 [87]	Houx P. Cognitive aging and health-related factors. Maastricht, Netherlands: Maastricht University, 1991:113–21.
Identical Forms	Mark the complex figure with the same from within a group of 5 different figures.	Lewerin, 2005 [79]	Dureman I, Sa¨lde H. Psykometriska och experimentalpsykologiska metoder för klinisk tilla¨mpning. (Psychometric and experimental psychological methods for clinical application.) Stockholm, Sweden: Almqvist & Wiksell, 1959 (in Swedish).
Motor Planning 2	Press a lit button out of 3 buttons as quickly as possible.	Eussen, 2006 [87]	Houx P. Cognitive aging and health-related factors. Maastricht, Netherlands: Maastricht University, 1991:113–21.
Reaction Time, Choice	Electronic reaction timer providing single challenges.	Dangour, 2015 [72]<br>Kwok, 2017 [74]	Cox BD, Huppert FA, Whichelow MJ. The Health and Lifestyle Survey: seven years on. Aldershot (United Kingdom): Dartmouth Publishing; 1993.

Test	Description	Author, year	References
Reaction Time, Simple	Electronic reaction timer providing multiple challenges.	Dangour, 2015 [72]<br>Kwok, 2017 [74]	Cox BD, Huppert FA, Whichelow MJ. The Health and Lifestyle Survey: seven years on. Aldershot (United Kingdom): Dartmouth Publishing; 1993.
Stroop Color-Word Test	Task 1: Read the color name given in black ink.<br>Task 2: Name the colored rectangles.<br>Task 3: Name the ink color of the printed words rather than the word	van der Zwaluw, 2014 [82]	Stroop J. Studies of interference in serial verbal reactions. J Exp Psychol 1935;18:643–662.
Symbol Digit Modalities Test	9 abstract symbols corresponding to numbers 1-9. Step 1: write correct number under each symbol during a 90 sec period. Step 2: say correct number to each symbol during a 90 sec period (oral version). Step 3: draw correct symbol under each number during a 90 sec period.	van der Zwaluw, 2014 [82]	Smith A. Symbol Digits Modalities Test. Los Angeles: Western Psychological Services; 1982.
Symbol Letter Modality	9 abstract symbols corresponding to numbers 1-9. Say correct number to each symbol during a 90 sec period (oral version).	Dangour, 2015 [72]	Smith A. Symbol letter modalities test. Revised manual. Los Angeles (CA): Western Psychological Services; 1982.
Trail Making Test	Part A: Connect randomly placed numbers (1-25) with a line as fast as possible in numeric order.<br>Part B: Connect randomly placed numbers (1-13) and letters (A-L) alternated with a line as fast as possible in numeric and alphabetical order.	Eussen, 2006 [87] (Part A)<br>McMahon, 2006 [80] (Part B)<br>van der Zwaluw, 2014 [82] (Part A)	Reitan R. Validity of the Trail Making Test as an indicator of organic brain damage. Percept Mot Skills 1958;8: 271–276.<br>Reitan RM. The relation of the Trail Making Test to organic brain damage. J Consult Psychol 1955;19:393-4.
Depression			
Beck Depression Inventory (BDI)	Patient self-reported 21-item questionnaire to measure severity of depression.	Ford, 2008 [78]	Beck AT, Ward CH, Mendelson M, et al. An inventory for measuring depression. Arch Gen Psychiatry 1961;4:561–571
Hamilton Depression Rating Scale (HDRS)	Clinician-administered questionnaire to measure severity of depressive symptoms. It consists of 17 items.	Kwok, 2020 [86]	Hamilton, M. (1967) Development of a rating scale for primary depressive illness. British Journal of Social and Clinical Psychology; 6(4):278-96.

Test	Description	Author, year	References
Kessler Psychological Distress Scale (K10)	Patient self-reported 10-item questionnaire to measure psychological distress (anxiety and depressive symptoms).	Christensen, 2011 [75]	Kessler, R., Andrews, G., Colpe, L., Hiripi, E., Mrockzek, D., Normand, S., et al., 2002. Short screening scales to monitor population prevalences and trends in non-specific psychological distress. Psychol. Med. 32 (6), 959–976.
Major Depression Inventory (MDI)	Patient self-reported 12-item questionnaire to measure DSM-IV and ICD-10 diagnosis of depression and severity of depression.	Hvas, 2004 [73]	Bech, P., Rasmussen, N.A., Olsen, L.R., Noerholm, V., Abildgaard, W., 2001. The sensitivity and specificity of the major depression inventory, using the present state examination as the index diagnostic validity. J. Affect. Disord. 66, 159–164.
Patient Health Questionnaire 9 (PHQ-9)	Patient self-reported 9-item questionnaire to measure presence and severity of depression.	Walker, 2010 [85]<br>Christensen, 2011 [75]	Spitzer, R., Kroenke, K., Williams, J., 1999. Validation and utility of a selfreport version of PRIME-MD—the PHQ primary care study. JAMA 282 (18), 1737–1744.
SF-12 Mental Component Summary (MCS) Score	Patient self-reported 6-item questionnaire (part of SF-12) to measure depressive disorders.	de Koning, 2016 [76]	Ware, J.; Kosinski, M.; Keller, S.D. A 12-Item Short-Form Health Survey: Construction of scales and preliminary tests of reliability and validity. Med. Care 1996, 34, 220–233.
Fatigue			
SF-36 Vitality	Patient self-reported 4-item questionnaire (part of SF-36) to measure vitality (energy level and fatigue).	Schlichtiger, 1996 [81]	Ware JE, Snow KK, Kosinski M, Gandek B (1993) Sf-36 health survey: manual and interpretation guide. The Health Institute, New England Medical Center, Boston

Supplement Table S3: Reasons for exclusion after full text analysis

Author, year	Reason for exclusion
Almeida, 2014	Patient population (depression)
Bjorkegren, 1999	No RCT
Brito, 2017	No outcomes of interest
Christen, 2018	No outcomes of interest
De Jager, 2012	Only subgroup analyses
Den Heijer, 2007	No outcomes of interest
Dhonukshe-Rutten, 2005	No outcomes of interest
Djokic, 2017	Multivitamin supplement
Ellis, 1973	High risk of bias
Flicker, 2006	No outcomes of interest
Garcia, 2004	Population n < 40
Hill, 2013	No outcomes of interest
Huang, 1999	Letter to the editor
Jiang B, 2013	Patient population (stroke)
Jiang Y, 2013	Congress abstract
Kang, 2008	Patient population (CVD)
Kurt, 2010	No outcomes of interest
Lewerin, 2003	No outcomes of interest
Lorenzl, 2008	No RCT
McMahon, 2007	No outcomes of interest
Miles, 2017	No outcomes of interest
Naurath, 1995	No outcomes of interest

**Author, year**	**Reason for exclusion**
Nct, 2010	Trial registration
Okereke, 2015	Patient population (CVD)
Oulhaj, 2016	Only subgroup analyses
Rietsema, 2014	Case report
Rosenberg, 2008	No RCT
Shahab-Ferdows, 2012	No outcomes of interest
Smith, 2010	No outcomes of interest
Stott, 2005	Patient population (CVD)
Swart, 2016	No outcomes of interest
Van Campen, 2019	No RCT
Van Der Zwaluw, 2013	Congress abstract
Van Uffelen, 2007	No outcomes of interest
Van Uffelen, 2010	Congress abstract
Van Wijngaarden, 2014	No outcomes of interest
Winkels, 2008	No outcomes of interest

Supplement Table S4: Additional patient characteristics at baseline of included studies

Author, year	Group	Participants, n	Female, n (%)	Mean age (SD), years	Mean MMSE (SD)	Mean Holotrans-cobalamin (SD), (pmol/L)	Mean MMA (SD), (µmol/L)	Mean homocysteine (SD), (µmol/L)
B12 alone								
Dangour, 2015 [72]	I	99	54 (54.5)	79.9 (3.5)	29.0 [28.0-29.0][a]	50.4 [38.2-68.3][a]	n.r.	15.9 [14.0-18.9][a]
	P	102	52 (51.0)	80.1 (3.7)	29.0 [28.0-29.0][a]	48.8 [39.8-62.9][a]	n.r.	16.3 [13.3-19.9][a]
Eussen, 2006 [87]	I	64	49 (76.6)	82.0 (5.0)	26.7 (3.1)	58.0 (21.0)	0.5 (0.4)	15.6 (6.6)
	P	65	51 (78.5)	82.0 (5.0)	26.8 (2.9)	70.0 (39.0)	0.5 (0.3)	15.8 (5.6)
Hvas, 2004 [73]	I	70	50 (71.4)	75.0 [19.0-92.0][b]	26.0 [n.r.][a]	n.r.	0.5 [0.3-2.0][a]	13.5 [5.3-46.6][a]
	P	70	48 (68.6)	74.0 [33.0-88.0][b]	27.0 [n.r.][a]	n.r.	0.5 [0.3-1.3][a]	12.8 [5.8-31.3][a]
Kwok, 2017 [74]	I	137	55 (40.0)	74.7 (4.0)	25.3 (3.8)	65.1 (28.6)	0.3 (0.2)	16.5 (5.4)
	P	134	58 (43.3)	75.8 (4.2)	25.2 (3.4)	66.8 (24.6)	0.2 (0.2)	17.1 (5.9)
B complex								
Christensen, 2011 [75]	I1[c]	109	72 (66.1)	65.8 (4.1)	n.r.	n.r.	n.r.	n.r.
	P1[c]	100	63 (63.0)	66.0 (4.2)	n.r.	n.r.	n.r.	n.r.
	I2	338	194 (57.4)	66.0 (4.4)	n.r.	n.r.	n.r.	n.r.
	P2	353	207 (58.6)	65.97 (4.2)	n.r.	n.r.	n.r.	n.r.
de Koning, 2016 [76]	I	1461	736 (50.4)	73.0 [69.0-78.0][a]	28.0 [27.0-29.0][a]	65.0 [48.0-86.0][a]	0.2 [0.2-0.3][a]	14.3 [13.0-16.5][a]
	P	1458	724 (49.7)	73.0 [69.0-78.0][a]	28.0 [27.0-29.0][a]	63.0 [45.0-84.0][a]	0.2 [0.2-0.3][a]	14.5 [13.0-16.7][a]

Author, year	Group	Participants, n	Female, n (%)	Mean age (SD), years	Mean MMSE (SD)	Mean Holotrans-cobalamin (SD), (pmol/L)	Mean MMA (SD), (µmol/L)	Mean homocysteine (SD), (µmol/L)
Eussen, 2006 [87]	I	66	49 (74.2)	83.0 (6.0)	26.7 (3.0)	68.0 (33.0)	0.4 (0.2)	14.5 (4.4)
	P	65	51 (78.5)	82.0 (5.0)	26.8 (2.9)	70.0 (39.0)	0.5 (0.3)	15.8 (5.6)
Ford, 2008 [78]	I	150	0 (0.0)	79.3 (2.7)	27.5 (1.8)	n.r.	n.r.	n.r.
	P	149	0 (0.0)	78.7 (2.7)	27.6 (1.9)	n.r.	n.r.	n.r.
Ford, 2010 [77]	I	150	0 (0.0)	79.3 (2.7)	27.5 (1.8)	n.r.	n.r.	14.0 (6.5)
	P	149	0 (0.0)	78.7 (2.7)	27.6 (1.9)	n.r.	n.r.	13.1 (3.8)
Kwok, 2020 [86]	I	138	51 (37.0)	76.9 (5.4)	26.0 (3.1)	85.5 (29.8)	n.r.	13.8 (3.4)
	P	141	62 (44.0)	78.0 (5.3)	25.7 (3.0)	85.8 (32.9)	n.r.	13.8 (3.0)
Lewerin, 2005 [79]	I	126	78 (62.0)	75.7 (4.7)	n.r.	n.r.	0.2 (0.1)	17.8 (5.5)
	P	69	39 (56.0)	75.6 (4.0)	n.r.	n.r.	0.2 (0.1)	16.1 (4.5)
McMahon, 2006 [80]	I	138	65 (52.0)	73.6 (5.8)	29.2 (1.0)	n.r.	n.r.	16.8 (5.4)
	P	138	47 (37.0)	73.4 (5.7)	29.2 (1.1)	n.r.	n.r.	16.3 (4.4)
Schlichtiger, 1996 [81]	I	110	76 (69.1)	73.3 (5.2)	n.r.	n.r.	n.r.	15.2 (5.2)
	P	103	74 (71.8)	73.3 (5.9)	n.r.	n.r.	n.r.	16.3 (6.0)
van der Zwaluw, 2014 [82]	I1	1461	736 (50.4)	73.0 [69.0-78.0][a]	28.0 [27.0-29.0][a]	65.0 [48.0-86.0][a]	0.2 [0.2-0.3][a]	14.3 [13.0-16.5][a]
	P1	1458	724 (49.7)	73.0 [69.0-78.0][a]	28.0 [27.0-29.0][a]	63.0 [45.0-84.0][a]	0.2 [0.2-0.3][a]	14.5 [13.0-16.7][a]

Author, year	Group	Participants, n	Female, n (%)	Mean age (SD), years	Mean MMSE (SD)	Mean Holotrans-cobalamin (SD), (pmol/L)	Mean MMA (SD), (µmol/L)	Mean homocysteine (SD), (µmol/L)
	I2[d]	425	181 (42.6)	n.r.	29.0 [27.0-30.0][a]	59.0 [46.0-78.0][a]	0.2 [0.2-0.3][a]	14.0 [12.9-16.2][a]
	P2[d]	431	176 (40.8)	n.r.	29.0 [28.0-30.0][a]	60.0 [42.0-80.0][a]	0.2 [0.2-0.3][a]	14.4 [12.9-16.4][a]
van Uffelen, 2008 [83]	I	90	34 (43.6)	75.4 (n.a.)	n.a.	n.r.	n.r.	n.r.
	P	89	33 (44.6)	74.9 (n.a.)	n.a.	n.r.	n.r.	n.r.
Walker, 2010 [85]	I	447	266 (59.5)	65.9 (4.3)	n.r.	n.r.	n.r.	9.6 (2.6)
	P	453	276 (60.9)	66.0 (4.2)	n.r.	n.r.	n.r.	9.8 (2.8)
Walker, 2012 [84]	I	447	266 (59.5)	65.9 (4.3)	n.r.	n.r.	n.r.	9.6 (2.6)
	P	453	276 (60.9)	66.0 (4.2)	n.r.	n.r.	n.r.	9.8 (2.8)

MMA, Methylmalonic Acid; MMSE, Mini-Mental State Examination; n.a., not applicable; n.r., not reported

[a] median [IQR]; [b] median [range]; [c] with antidepressant; [d] extensive cognitive tests subsample

Supplement Table S5: Study characteristics

Author, year	Study location, country	Inclusion	Exclusion
B12 alone			
Dangour, 2015 [72]	General practices (n=7) in South East England that were members of the Medical Research Council General Practice Research Framework or the National Institute of Health Research Primary Care Research Network, England	• Mini-Mental State Examination score ≥24<br>• moderate vitamin B-12 deficiency who did not have anemia (serum vitamin B-12 concentrations ≥107 and <210 pmol/L [Beckman Coulter assay] and hemoglobin concentrations ≥110 g/L for women and ≥120 g/L for men)	• current consumption of vitamin B-12 supplements<br>• received a vitamin B-12 injection in the previous 6 mo<br>• very-low vitamin B-12 concentrations (<107 pmol/L, which is a cutoff typically used for deficiency; Beckman Coulter assay, Beckman Coulter Inc.)<br>• anemia (hemoglobin concentration <110 g/L for women and <120 g/L for men)<br>• diabetes, dementia, epilepsy, alcohol addiction, pacemakers and implanted metallic devices, previous pernicious anemia<br>• residents of nursing homes
Eussen, 2006 [87]	n.r., the Netherlands	• mild vitamin B-12 deficiency was defined as<br>1) a serum vitamin B-12 concentration between 100 and 200 pmol/L, or<br>2) a serum vitamin B-12 concentration between 200 and 300 pmol/L, a plasma MMA concentration ≥0.32 mol/L, and a serum creatinine concentration ≤120 mol/L, the latter intended to exclude severe impairment of renal function<br>• Mini-Mental State Examination (MMSE) ≥19 points	• history of cobalamin deficiency, use of cobalamin (>50 µg/d) or folic acid (>200 µg/d) supplementation or injections<br>• surgery or diseases of the stomach or small intestine, anemia, dementia, life-threatening diseases, or severe hearing or visual problems<br>• ingested <90% of the placebo or if MMSE was <19 points (after 2 wk run-in period)
Hvas, 2004 [73]	Aarhus University Hospital, Denmark	• mildly to modestly increased P-MMA (0.40–2.00 umol/L)<br>• not previously treated with vitamin B12	• P-MMA >2.00 umol (n=5)<br>• blood hemoglobin ≤6.0 mmol/L and erythrocyte mean cell volume ≥110 fL (n=0)<br>• blood hemoglobin ≤6.0 mmol/L and plasma ferritin ≤10 ug/L (males) or ≤8 ug/L (females; n=0)<br>• plasma thyroid-stimulating hormone ≥4.1 mIU/L (n=6)<br>• plasma creatinine >120 umol/L (females) or >133 mmol/L (males; n=41)<br>• incapacity to give informed consent, non-Danish speaking

Author, year	Study location, country	Inclusion	Exclusion
			• life-threatening disease, hypersensitivity to cyanocobalamin, treatment with anticoagulants, Leber hereditary optic neuropathy, tobacco-alcohol amblyopia, tropical atoxic neuropathy, deafness or severe visual impairment, severe aphasia, current participation in another clinical trial
Kwok, 2017 [74]	Medical/diabetic clinics in Prince of Wales Hospital (PWH) and the seven Family Medicine/general outpatient clinics in the New Territories East cluster in Hong Kong, Hong Kong	• diabetic people aged 70 years or older with plasma vitamin B12 150-300 pmol/L	• dementia or peripheral neuropathy or anemia, disabling stroke, renal failure, clinical depression, positive intrinsic factor antibody<br>• current consumption of vitamin B12 supplements or centrally acting medications<br>• no family member who could reliably inform on cognitive functioning (personal contact at least once a week)
B complex			
Christensen, 2011 [75]	Australia	• elevated psychological distress as assessed by the K10 with scores 16 or greater (scores <16 indicate no or low levels of psychological distress)<br>• did not engage in physical activity at public health recommended levels as indicated by International Physical Activity Questionnaire (IPAQ) scores<br>• did not take folic acid, vitamin B12 or vitamin B complex supplements<br>• had no history of dementia, bipolar disorder or current suicide risk<br>• had competent literacy skills<br>• did not have a medical condition that would contraindicate exercise or folic acid use	• high likelihood of a depressive disorder with K10 scores of 30 or greater<br>• low levels of red cell folate (<250 nmol/l) and vitamin B12 (<130 nmol/l)<br>• abnormal thyroid-stimulating hormone levels (0.35–5.0 mu/l)
de Koning, 2016 [76]	Erasmus Medical Center (EMC, Rotterdam), VU University Medical Center (VUmc,	• men and women aged 65 years and older<br>• independently living or institutionalized<br>• compliance for tablet intake of > 85% 4-6 weeks prior to start of the trial	• immobilization: being bedridden or wheelchair bound<br>• cancer diagnosis within the last 5 year, except skin cancer as basal cell carcinoma and squamous cell carcinoma<br>• serum creatinine level >150 µmol/L

Author, year	Study location, country	Inclusion	Exclusion
	Amsterdam) and Wageningen University (WU, Wageningen), Netherlands	• competent to make own decisions<br>• elevated homocysteine level (≥12 µmol/L and ≤50 µmol/L)	• current or recent (<4 months) use of supplements with very high doses of vitamin B12 (intramuscular injections) or folic acid (>300 µg)<br>• participation in other intervention studies
Eussen, 2006 [87]	*See at B12 alone*	*See at B12 alone*	*See at B12 alone*
Ford, 2008 [78]	Perth, Western Australia	• men aged 75 years and older<br>• being treated for, or had a history of, hypertension	• Beck Depression Inventory (BDI) score of 18 or higher<br>• significant cognitive impairment as evidenced by a Mini-Mental State Examination (MMSE) score of less than 24 points<br>• illness deemed likely to cause severe disability or death within 12 months (for example, metastatic cancer, Parkinson's disease, or history of stroke)<br>• living in residential care facilities<br>• non–English speakers<br>• already taking vitamin B supplements
Ford, 2010 [77]	Perth, Western Australia	• men aged 75 years and older<br>• being treated for, or had a history of, hypertension	• Beck Depression Inventory (BDI) score of 18 or higher<br>• significant cognitive impairment as evidenced by a Mini-Mental State Examination (MMSE) score of less than 24 points<br>• illness deemed likely to cause severe disability or death within 12 months (for example, metastatic cancer, Parkinson's disease, or history of stroke)<br>• living in residential care facilities<br>• non–English speakers<br>• already taking vitamin B supplements
Kwok, 2020 [86]	Specialist medical outpatient clinics in Prince of Wales Hospital, Hong Kong, China	• people aged 65 years or more<br>• Montreal cognitive assessment (MoCA) test (Hong Kong version) score lower than 22	• serum homocysteine less than 10 mmol/L<br>• dementia, peripheral neuropathy, clinical depression, renal failure, anemia, disabling stroke<br>• receiving centrally acting medications or B vitamins supplementation

Author, year	Study location, country	Inclusion	Exclusion
			• no reliable caregiver or family member who was able to regularly inform on cognitive functioning (at least one personal contact each week)
Lewerin, 2005 [79]	Göteborg, Sweden	• participants recruited from a previous study (Augustsson et al. 1994)<br>• men and women aged 70 years and older<br>• living in a defined central area of the city of Gothenburg (Johanneberg)	• had taken any vitamin supplements during the last 3 months<br>• had taken pharmacological doses of vitamin B12, folic acid and/or vitamin B6 during the last 3 years
McMahon, 2006 [80]	Dunedin, New Zealand	• volunteers 65 years of age or older	• suspected dementia<br>• taking medications known to interfere with folate metabolism (e.g., oral hypoglycemic agents or antiepileptic agents)<br>• taking vitamin supplements containing folic acid, vitamin B12, or vitamin B6<br>• being treated for depression<br>• diabetes<br>• history of stroke or transient ischemic attacks
Schlichtiger, 1996 [81]	licensed general practitioners, internists and neurologists, Germany	• men and women 65-85 years of age<br>• lost of vitality that led to reduced quality of life<br>• Nürnberger Alltags-Beobachtungs-Skala (NAB) <22<br>• SF-36 subscale "vitality" between ≥12 and ≤18	• severe psychiatric diseases<br>• Morbus Parkinson<br>• psychiatric depressions (Self-Rating Depression Scale (SDS) >47)<br>• dementia (cognitive minimal screening <13)<br>• lower intelligence (IQ≤85)<br>• rheumatic, muscular, nerval or joint diseases causing strong restriction of motion<br>• malignant diseases<br>• uncontrolled diabetes mellitus<br>• decompensated heart failure<br>• unstable arterial hypertension
van der Zwaluw, 2014 [82]	Erasmus Medical Center (EMC, Rotterdam), VU University Medical	• men and women aged 65 years and older<br>• independently living or institutionalized	• immobilization: being bedridden or wheelchair bound<br>• cancer diagnosis within the last 5 year, except skin cancer as basal cell carcinoma and squamous cell carcinoma

Author, year	Study location, country	Inclusion	Exclusion
	Center (VUmc, Amsterdam) and Wageningen University (WU, Wageningen), Netherlands	• compliance for tablet intake of > 85% 4-6 weeks prior to start of the trial<br>• competent to make own decisions<br>• elevated homocysteine level (≥12 µmol/L and ≤50 µmol/L)	• serum creatinine level >150 µmol/L<br>• current or recent (<4 months) use of supplements with very high doses of vitamin B12 (intramuscular injections) or folic acid (>300 µg)<br>• participation in other intervention studies
van Uffelen, 2008 [83]	Alkmaar, Netherlands	• independently living elderly aged between 70 and 80 years with mild cognitive impairment (MCI) defined as follows:<br>- memory complaints (answer "yes" to question "do you have memory complaints", or at least twice answering "sometimes" on the Strawbridge cognition scale)<br>- objective memory impairment (10 word learning test (WLT) delayed recall ≤5 and percentage savings ≤100)<br>- normal general cognitive function/absence of dementia (telephone interview for cognitive status (TICS) ≥19 and mini mental state examination (MMSE) ≥24)<br>- intact daily functioning (no report of disability in activities of daily living on Groningen activity restriction scale (GARS), except on the item "taking care of feet and toe nails")<br>• being able to perform moderate intensity physical activity without making use of walking devices—for example, a rollator or a walking frame<br>• not using vitamin supplements/vitamin injections/drinks with folic acid, vitamins B-12 and B-6, comparable to the vitamin supplement given in the intervention<br>• not suffering from epilepsy, multiple sclerosis, Parkinson's disease, kidney disorder requiring haemodialysis, psychiatric impairment<br>• not suffering from depression as measured by the geriatric depression scale (GDS, cut off ≤5)	• see inclusion criteria

Author, year	Study location, country	Inclusion	Exclusion
		• not using medication for rheumatoid arthritis or psoriasis which interfered with the vitamin supplement<br>• no alcohol abuse (men <21 drinks a week, women <15 drinks a week)<br>• not currently living in a nursing home or on a waiting list for a nursing home"	
Walker, 2010 [85]	Australia	• elevated psychological distress as assessed by the K10 with scores 16 or greater (scores <16 indicate no or low levels of psychological distress)<br>• did not engage in physical activity at public health recommended levels as indicated by International Physical Activity Questionnaire (IPAQ) scores<br>• did not take folic acid, vitamin B12 or vitamin B complex supplements<br>• had no history of dementia, bipolar disorder or current suicide risk<br>• had competent literacy skills<br>• did not have a medical condition that would contraindicate exercise or folic acid use	• high likelihood of a depressive disorder with K10 scores of 30 or greater<br>• low levels of red cell folate (<250 nmol/l) and vitamin B12 (<130 nmol/l)<br>• abnormal thyroid-stimulating hormone levels (0.35–5.0 mu/l)
Walker, 2012 [84]	Australia	• elevated psychological distress as assessed by the K10 with scores 16 or greater (scores <16 indicate no or low levels of psychological distress)<br>• did not engage in physical activity at public health recommended levels as indicated by International Physical Activity Questionnaire (IPAQ) scores<br>• did not take folic acid, vitamin B12 or vitamin B complex supplements<br>• had no history of dementia, bipolar disorder or current suicide risk<br>• had competent literacy skills<br>• did not have a medical condition that would contraindicate exercise or folic acid use	• high likelihood of a depressive disorder with K10 scores of 30 or greater<br>• low levels of red cell folate (<250 nmol/l) and vitamin B12 (<130 nmol/l)<br>• abnormal thyroid-stimulating hormone levels (0.35–5.0 mu/l)

Supplement Table S6: GRADE summary of findings

Certainty assessment							№ of patients		Effect		Certainty	Importance
**№ of studies**	**Study design**	**Risk of bias**	**Inconsistency**	**Indirectness**	**Imprecision**	**Other considerations**	**Intervention**	**Placebo**	**Relative (95% CI)**	**Absolute (95% CI)**		
Cognitive Executive												
10	randomised trials	not serious [a]	not serious [b]	serious [c]	not serious [d]	none	1178	1147	-	SMD **0.06 SD higher** (0.02 lower to 0.14 higher)	⨁⨁⨁◯ MODERATE	CRITICAL
Cognitive Memory												
12	randomised trials	not serious [e]	not serious [f]	serious [c]	not serious [d]	none	2559	2523	-	SMD **0.03 SD higher** (0.01 lower to 0.07 higher)	⨁⨁⨁◯ MODERATE	CRITICAL
Cognitive Global												
7	randomised trials	not serious	not serious [g]	serious [c]	not serious [d]	none	1807	1814	-	SMD **0.06 SD higher** (0 to 0.12 higher)	⨁⨁⨁◯ MODERATE	IMPORTANT
Cognitive Speed												
8	randomised trials	not serious [h]	not serious [i]	serious [c]	not serious [d]	none	907	876	-	SMD **0.08 SD lower** (0.18 lower to 0.01 higher)	⨁⨁⨁◯ MODERATE	IMPORTANT
Depression												
6	randomised trials	not serious [j]	not serious [k]	serious [c]	not serious [d]	none	2448	2487	-	SMD **0.05 SD lower** (0.15 lower to 0.05 higher)	⨁⨁⨁◯ MODERATE	IMPORTANT

CI: Confidence interval; **SMD:** Standardised mean difference

Explanations

a. 1/9 had some concerns reported due to "Deviations from intended interventions". 2/9 had some concerns reported due to "Selection of the reported result".

b. Pooled I-squared=13.1%, p=0.82. Good overlap of confidence intervals.

c. duration of intervention varied across trials; different tests/instruments were used for the same outcome domain; different vitamin intervention (B12 alone or B complex) and application doses

d. Confidence interval is within +-0.2 (Cochrane Handbook, Version 6.1, 2020, Chapter 15.5.3.1), commonly used as minimum thresholds for small effects

e. 1/11 had some concerns reported due to "Deviations from intended interventions". 2/11 had some concerns reported due to "Selection of the reported result".

f. Pooled I-squared=0.0%, p=0.98. Good overlap of confidence intervals.

g. Pooled I-squared=0.0%, p=0.95. Good overlap of confidence intervals.

h. 1/7 had some concerns reported due to "Deviations from intended interventions". 2/7 had some concerns reported due to "Selection of the reported result".

i. Pooled I-squared=13.9%, p=0.88. Good overlap of confidence intervals.

j. 1/6 had some concerns reported due to "Missing outcome data".

k. Pooled I-squared=22.5%, p=0.50. Good overlap of confidence intervals.

Supplement Table S7: Meta-regression models for the analysis of the cognitive executive and memory subdomains

Outcome subdomain	Term	Estimate	95% CI LB	95% CI UB	p-value
With baseline vitamin B12 serum level					
Cognitive Executive	Intercept	0.14	-2.572	2.851	0.91
	Baseline vitamin B12 serum level	0	-0.005	0.005	0.98
	Vitamin intervention B complex	-0.036	-0.583	0.511	0.89
	Duration of treatment (in weeks)	-0.002	-0.016	0.012	0.75
	Average bioavailable daily dose (in mcg)	0.003	-0.198	0.204	0.97
	Population "No MCI to MCI"	-0.252	-0.800	0.295	0.35
	Duration * daily dose	0	-0.002	0.002	0.71
Cognitive Memory	Intercept	-0.39	-1.003	0.223	0.2
	Baseline vitamin B12 serum level	0	-0.002	0.002	0.95
	Vitamin intervention B complex	0.189	-0.055	0.433	0.12
	Duration of treatment (in weeks)	0.002	-0.001	0.005	0.11
	Average bioavailable daily dose (in mcg)	0.02	-0.008	0.047	0.15
	Population "No MCI to MCI"	0.213	-0.035	0.461	0.09
	Duration * daily dose	0	-0.001	0	0.23
Without baseline vitamin B12 serum level					
Cognitive Executive	Intercept	0.103	-0.946	1.153	0.84
	Vitamin intervention B complex	-0.015	-0.470	0.440	0.95
	Duration of treatment (in weeks)	-0.001	-0.010	0.007	0.73
	Average bioavailable daily dose (in mcg)	0.018	-0.105	0.140	0.77
	Population "No MCI"	-0.077	-0.383	0.228	0.61

	Population “No MCI to MCI”	-0.354	-0.890	0.181	0.19
	Duration * daily dose	0	-0.001	0.001	0.66
Cognitive Memory	Intercept	-0.399	-0.832	0.033	0.07
	Vitamin intervention B complex	0.189	-0.048	0.425	0.11
	Duration of treatment (in weeks)	0.002	-0.001	0.005	0.12
	Average bioavailable daily dose (in mcg)	0.019	-0.008	0.046	0.16
	Population “No MCI”	0.041	-0.194	0.277	0.72
	Population “No MCI to MCI”	0.253	-0.076	0.582	0.13
	Duration * daily dose	0	-0.001	0	0.22

CI: confidence interval; LB: lower bound; UB: upper bound

Supplement Figure S1: Quality assessment of included studies

	Author	Year	Randomization process	Deviations from intended interventions	Missing outcome data	Measurement of the outcome	Selection of the reported result	Overall bias
B12 alone	Dangour [72]	2015	Low risk	Low risk	Low risk	Low risk	Low risk	Low risk
B12 alone	Eussen [87]	2006	Low risk	Low risk	Low risk	Low risk	Some concerns	Some concerns
B12 alone	Hvas [73]	2004	Low risk	Low risk	Low risk	Low risk	Low risk	Low risk
B12 alone	Kwok [74]	2017	Low risk	Low risk	Low risk	Low risk	Low risk	Low risk
B12 alone	Ellis* [71]	1973	Some concerns	Low risk	High risk	Low risk	Some concerns	High risk
B complex	Christensen [75]	2011	Low risk	Low risk	Low risk	Low risk	Low risk	Low risk
B complex	de Koning [76]	2016	Low risk	Low risk	Low risk	Low risk	Low risk	Low risk
B complex	Eussen [87]	2006	Low risk	Low risk	Low risk	Low risk	Some concerns	Some concerns
B complex	Ford [78]	2008	Low risk	Low risk	Some concerns	Low risk	Low risk	Some concerns
B complex	Ford [77]	2010	Low risk	Low risk	Low risk	Low risk	Low risk	Low risk
B complex	Kwok [86]	2020	Low risk	Low risk	Low risk	Low risk	Low risk	Low risk
B complex	Lewerin [79]	2005	Low risk	Some concerns	Low risk	Low risk	Some concerns	Some concerns
B complex	McMahon [80]	2006	Low risk	Low risk	Low risk	Low risk	Low risk	Low risk
B complex	Schlichtiger [81]	1996	Some concerns	Low risk	Low risk	Low risk	Some concerns	Some concerns
B complex	van der Zwaluw [82]	2014	Low risk	Low risk	Low risk	Low risk	Low risk	Low risk
B complex	von Uffelen [83]	2008	Low risk	Low risk	Low risk	Low risk	Low risk	Low risk
B complex	Walker [85]	2010	Low risk	Low risk	Low risk	Low risk	Low risk	Low risk
B complex	Walker [84]	2012	Low risk	Low risk	Low risk	Low risk	Low risk	Low risk

Low risk
Some concerns
High risk

* = not included in the analysis

Supplement Figure S2: Funnel plots for cognitive function subdomains and depression domain

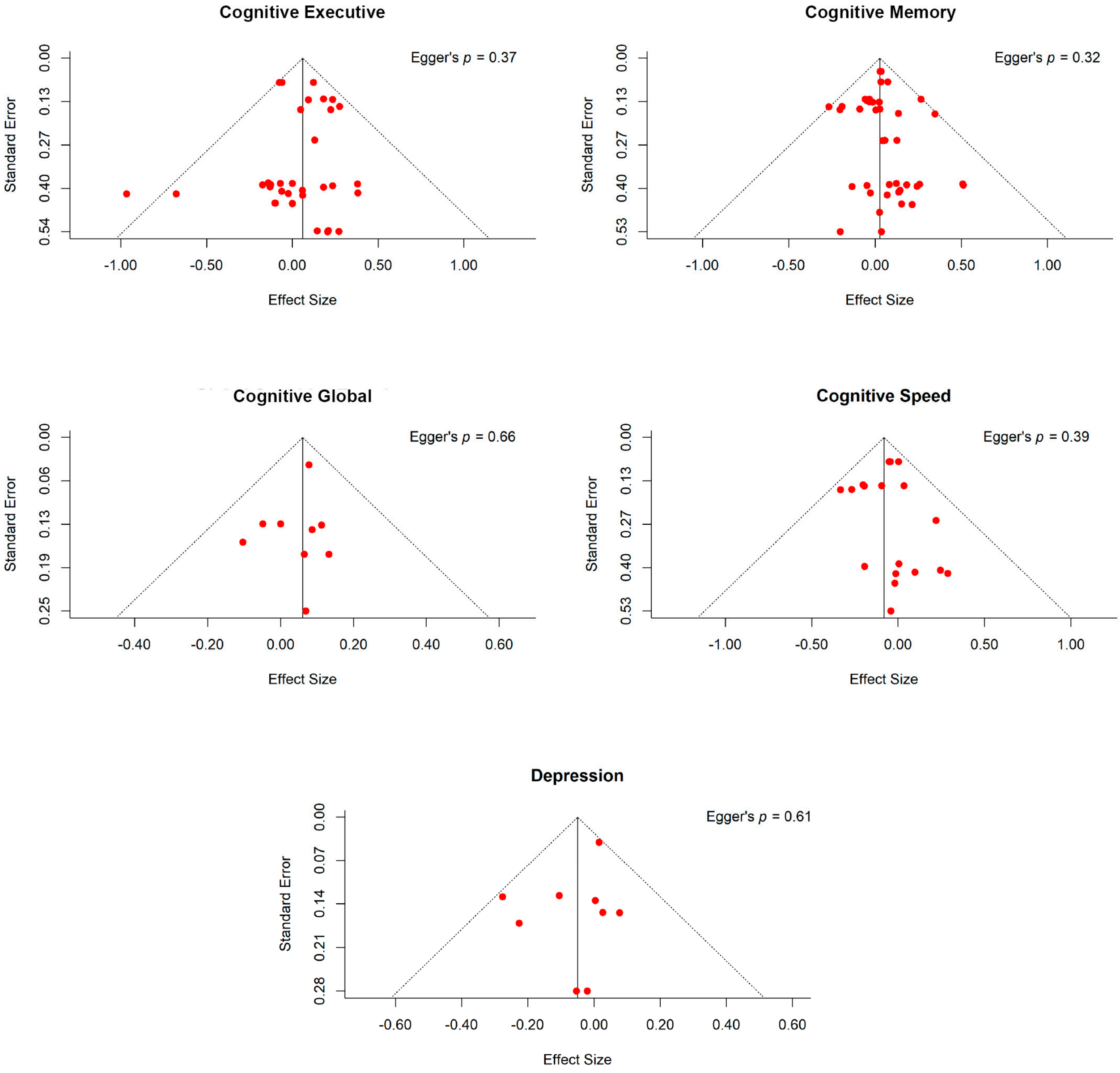

Each red circle corresponds to a clinical test outcome. The diagonal lines show the expected 95% confidence intervals around the summary estimate.

Supplement Figure S3: Forrest plot for effects on cognitive global function

Author (Year)	Outcome Measurement	N Intervention	N Placebo	Weight	Effect Size [95% CI]
B12 Alone					
Hvas (2004) [73]	CAMCOG	65	69	3.4%	0.13 [−0.20, 0.46]
Hvas (2004) [73]	MMSE	65	69	3.4%	0.06 [−0.27, 0.40]
Kwok (2017) [74]	Neuropsychological Test Battery (Total)	87	87	4.3%	−0.10 [−0.40, 0.19]
Subtotal (Heterogeneity: I^2 = 6.3%, τ^2 = 0.0018, p = 0.55)					0.02 [−0.17, 0.21]
B12 Complex					
Ford (2010) [77]	ADAS (Cognitive Subscale)	127	128	6.3%	−0.05 [−0.29, 0.20]
Ford (2010) [77]	MMSE	127	128	6.3%	0.00 [−0.25, 0.25]
Kwok (2020) [86]	CDR (Sum of Boxes)	123	126	6.1%	0.11 [−0.14, 0.36]
McMahon (2006) [80]	MMSE	127	126	1.6%	0.07 [−0.42, 0.56]
van der Zwaluw (2014) [82]	MMSE	1278	1278	63.0%	0.08 [−0.00, 0.16]
Walker (2012) [84]	TICS−M (Total Cognitive Functioning Score)	447	453	5.5%	0.09 [−0.17, 0.35]
Subtotal (Heterogeneity: I^2 = 0.0%, τ^2 = 0.0000, p = 0.93)					0.07 [0.00, 0.13]
Pooled					
RE model (Heterogeneity: I^2 = 0.0%, τ^2 = 0.0000, p = 0.95)					0.06 [−0.00, 0.12]

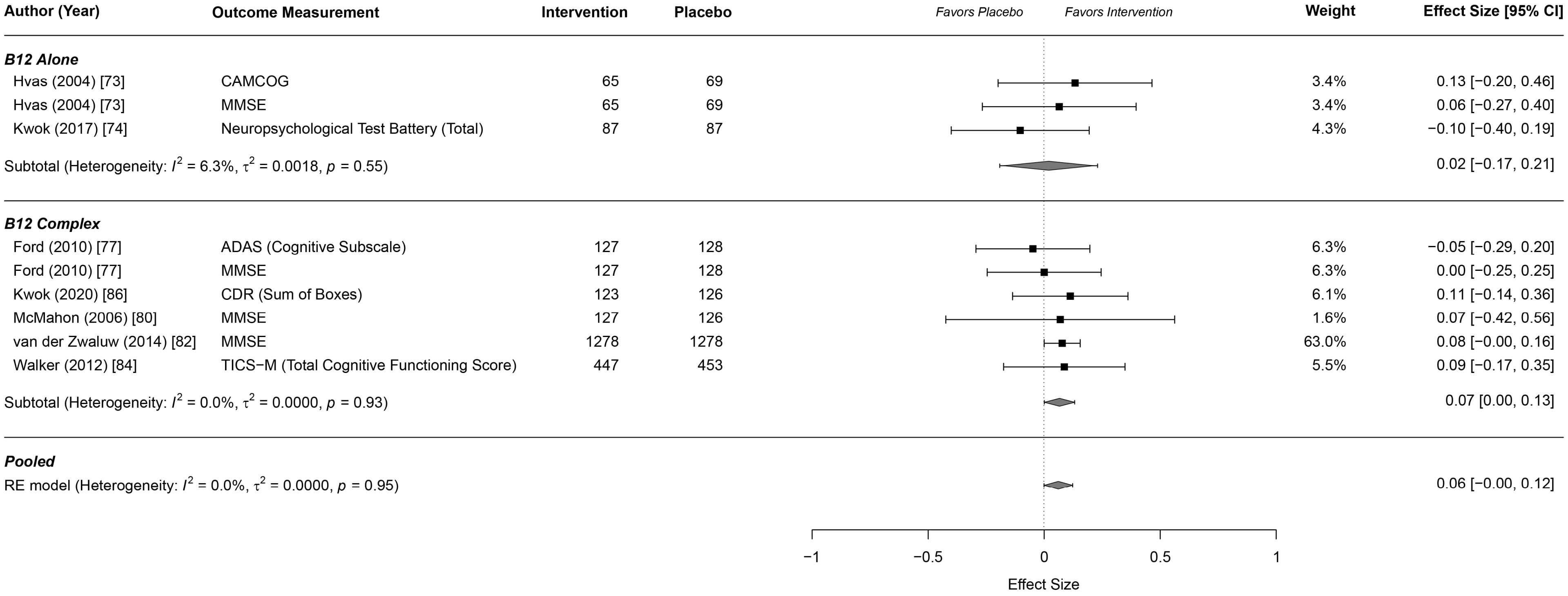

CAMCOG: Cambridge Cognitive Examination; CDR: Clinical Dementia Rating; MMSE: Mini-Mental State Examination; TICS-M: Telephone Interview for Cognitive Status Modified

Supplement Figure S4: Forrest plot for effects on cognitive speed function

Author (Year)	Outcome Measurement	N Intervention	N Placebo	Weight	Effect Size [95% CI]
B12 Alone					
Dangour (2015) [72]	Reaction Time (Choice)	91	93	5.69%	0.03 [−0.25, 0.32]
Dangour (2015) [72]	Reaction Time (Simple)	91	93	5.68%	−0.10 [−0.39, 0.19]
Dangour (2015) [72]	Symbol Letter Modality	91	93	5.66%	−0.20 [−0.49, 0.09]
Eussen (2006) [87]	Finger Tapping	48	50	0.81%	0.10 [−0.71, 0.90]
Eussen (2006) [87]	Motor Planning 2	48	50	0.83%	0.24 [−0.55, 1.04]
Eussen (2006) [87]	Trail Making Test (Part A)	53	56	0.91%	0.00 [−0.75, 0.76]
Kwok (2017) [74]	Reaction Time (Simple) & Reaction Time (Choice)	92	100	5.65%	−0.20 [−0.49, 0.08]
Subtotal (Heterogeneity: I^2 = 0.0%, τ^2 = 0.0000, p = 0.83)					−0.10 [−0.23, 0.04]
B12 Complex					
Eussen (2006) [87]	Finger Tapping	44	50	0.79%	−0.01 [−0.83, 0.80]
Eussen (2006) [87]	Motor Planning 2	44	50	0.79%	0.29 [−0.53, 1.10]
Eussen (2006) [87]	Trail Making Test (Part A)	48	56	0.88%	−0.19 [−0.97, 0.58]
Lewerin (2005) [79]	Digit Symbol	113	62	4.90%	−0.27 [−0.58, 0.04]
Lewerin (2005) [79]	Identical Forms	115	61	4.87%	−0.33 [−0.65, −0.02]
McMahon (2006) [80]	Trail Making Test (Part B)	127	126	2.05%	0.22 [−0.28, 0.72]
van der Zwaluw (2014) [82]	SCWT (Mean 1 and 2)	358	373	19.77%	−0.05 [−0.20, 0.09]
van der Zwaluw (2014) [82]	Symbol Digit Modalities Test	358	373	19.77%	0.00 [−0.14, 0.15]
van der Zwaluw (2014) [82]	Trail Making Test (Part A)	358	373	19.77%	−0.05 [−0.19, 0.10]
van Uffelen (2008) [83]	DSST (Men)	43	38	0.69%	−0.02 [−0.89, 0.85]
van Uffelen (2008) [83]	DSST (Women)	28	29	0.49%	−0.04 [−1.08, 1.00]
Subtotal (Heterogeneity: I^2 = 43.4%, τ^2 = 0.0149, p = 0.68)					−0.08 [−0.25, 0.09]
Pooled					
RE model (Heterogeneity: I^2 = 13.8%, τ^2 = 0.0037, p = 0.88)					−0.08 [−0.18, 0.01]

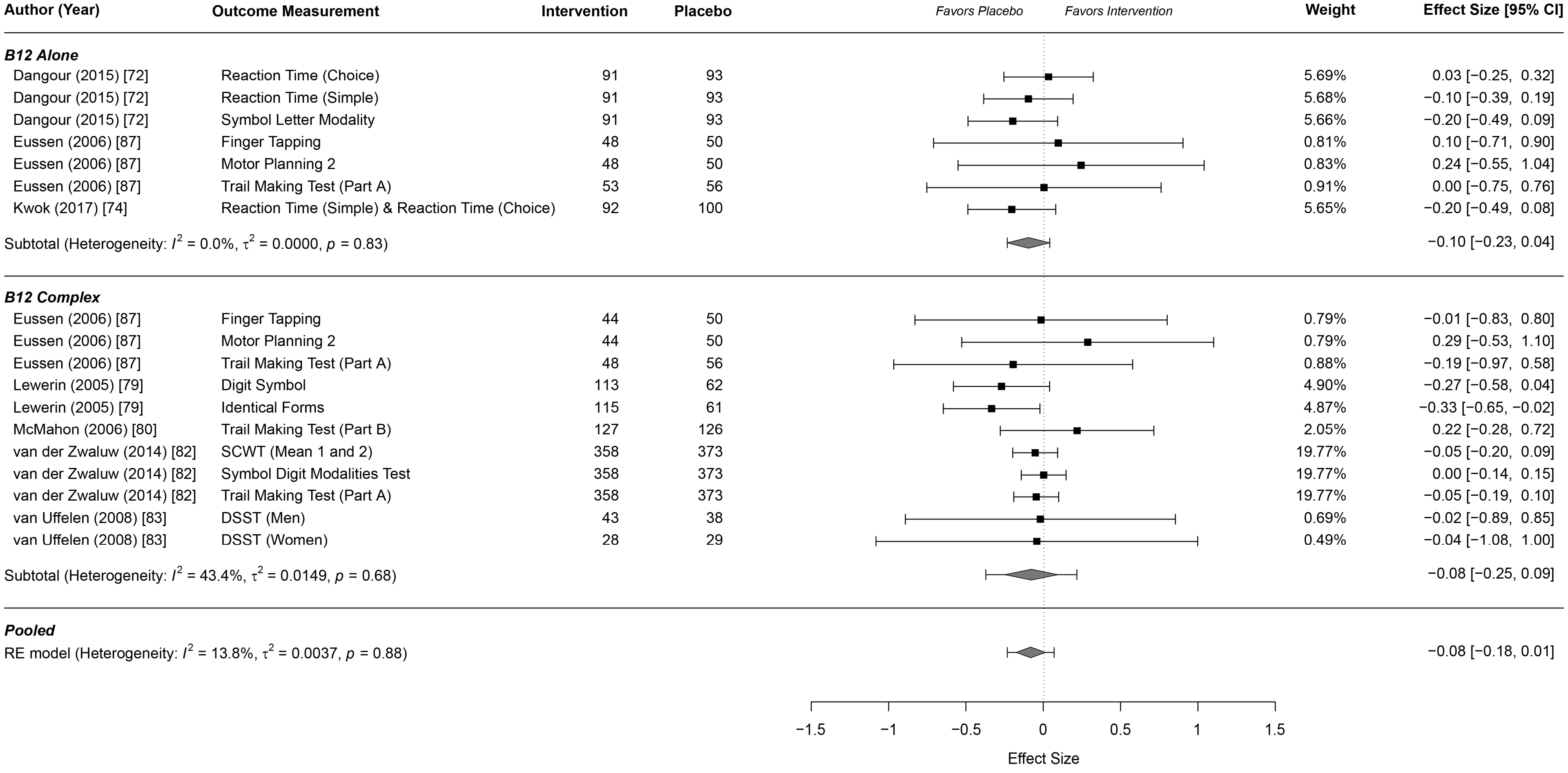

DSST: Digit Symbol Substitution Test; SCWT: Stroop Color-Word Test
